# Cytogenetic and molecular characterization of a recombinant X chromosome in a family with a severe neurologic phenotype and macular degeneration

**DOI:** 10.1186/s13039-015-0164-1

**Published:** 2015-08-01

**Authors:** Pamela Magini, Monica Poscente, Simona Ferrari, Manuela Vargiolu, Elena Bacchelli, Claudio Graziano, Anita Wischmeijer, Daniela Turchetti, Elisabetta Malaspina, Valentina Marchiani, Duccio Maria Cordelli, Emilio Franzoni, Giovanni Romeo, Marco Seri

**Affiliations:** U.O. Genetica Medica, Policlinico Sant’Orsola-Malpighi, DIMEC, Università di Bologna, via Massarenti, 9, Bologna, 40138 Italy; S.S.V.D. Biologia Molecolare, Citogenetica, Citomorfologia Ematica e Vaginale, Ospedale Belcolle, Viterbo, Italy; U.O. Genetica Medica, AOU di Bologna, Policlinico S. Orsola-Malpighi, Bologna, 40138 Italy; Centro Interdipartimentale per la Ricerca Industriale Scienze della Vita e Tecnologie per la Salute, Università di Bologna, Bologna, Italy; Dipartimento di Farmacia e Biotecnologie, Università di Bologna, Bologna, Italy; S.S.D. Genetica Clinica, Arcispedale S. Maria Nuova-Istituto di Ricovero e Cura a Carattere Scientifico, Reggio Emilia, Italy; U.O. Neuropsichiatria Infantile, Policlinico Sant’Orsola-Malpighi, DIMEC, Università di Bologna, Bologna, Italy

**Keywords:** *MECP2* duplication, Xq28 disomy, Recombinant X chromosome, Macular degeneration

## Abstract

**Background:**

Duplications of *MECP2* gene in males cause a syndrome characterized by distinctive clinical features, including severe to profound mental retardation, infantile hypotonia, mild dysmorphic features, poor speech development, autistic features, seizures, progressive spasticity and recurrent infections. Patients with complex chromosome rearrangements, leading to Xq28 duplication, share most of the clinical features of individuals with tandem duplications, in particular neurologic problems, suggesting a major pathogenetic role of *MECP2* overexpression.

**Results:**

We performed cytogenetic and molecular cytogenetic studies in a previously described family with affected males showing congenital ataxia, late-onset progressive myoclonic encephalopathy and selective macular degeneration. Microsatellite, FISH and array-CGH analyses identified a recombinant X chromosome with a deletion of the PAR1 region, encompassing *SHOX*, replaced by a duplicated segment of the Xq28 terminal portion, including *MECP2*.

**Conclusions:**

Our report describes the identification of the actual genetic cause underlying a severe syndrome that previous preliminary analyses erroneously associated to a terminal Xp22.33 region. In the present family as well as in previously reported patients with similar rearrangements, the observed neurologic phenotype is ascribable to *MECP2* duplication, with an undefined contribution of the other involved genes. Maculopathy, presented by affected males reported here, could be a novel clinical feature associated to Xq28 disomy due to recombinant X chromosomes, but at present the underlying pathogenetic mechanism is unknown and this potential clinical correlation should be confirmed through the collection of additional patients.

## Introduction

Large duplications of the X chromosome long arm are rare, usually involve the distal Xq27-qter region, and cause a severe phenotype in males, while most carrier females are phenotypically normal, as a consequence of a skewed inactivation of the abnormal chromosome [1, 2]. With the clinical application of array-CGH, Xq28 microduplications, including *MECP2* gene, emerged as one of the most common genomic rearrangements in males with developmental delay [3].

The majority of *MECP2* duplications are intrachromosomal and represent non-recurrent events, probably mediated by fork stalling and template switching (FoSTeS) mechanism; more rarely, they can arise from complex rearrangements, resulting in the translocation of the duplicated segment to the short arm of the X chromosome, to an autosome or to the Y chromosome [4–6].

In 1992, Bertini et al. described a family with a severe X-linked phenotype characterized by congenital ataxia with generalized hypotonia, psychomotor retardation, late-onset progressive myoclonic encephalopathy, selective macular degeneration and recurrent bronchopulmonary infections. Three-generations linkage analysis revealed a candidate disease-associated locus on Xp22.33-pter region [7, 8].

Here, we describe the genetic studies performed in the above mentioned affected family that led to the identification of a recombinant X chromosome (qter- > Xq28::Xp22.33- > qter) causing the severe clinical phenotype observed in affected males.

## Case presentation

A detailed clinical description of the family is available in the paper of Bertini et al. [7].

**Fig. 1:**
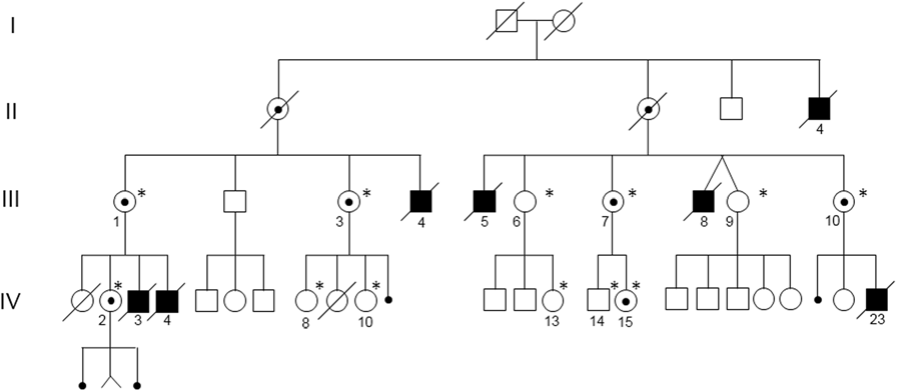
Family pedigree. Affected males (*filled symbols*) and females carrying the recombinant X chromosome (*circles with dots*) are reported. Family members studied through microsatellite analysis are indicated by an *asterisk*

Males II4, III4, III5, III8 and IV3 (Fig. 1) presented generalized hypotonia at birth and died in infancy because of severe gastroenteritis (II4) and bronchopneumonia (III4, III5, III8 and IV3).

Affected males IV4 and IV23 survived longer, developing additional clinical features.

### Patient IV4

This patient, born in 1979 from non-consanguineous parents, presented neonatal generalized hypotonia and delayed motor development. He could walk with support at 4 years, had poor language with dysarthric speech, tonic seizures and recurrent bronchopneumonia. Physical and neurological examinations revealed growth delay (height < 3rd centile, head circumference < 3rd centile and weight around the 3rd centile), severe trunk and limb ataxia, dysmetria, hypoactive deep tendon reflexes, stereotyped movements and low response to external stimuli. In the following years, the clinical course worsened with the occurrence of frequent (weekly) intractable seizures and visual, motor and intellectual deterioration leading to blindness, generalized muscular atrophy and severe hypotonia, with frequent segmental myoclonic jerks. Fundoscopy and brain CT-scan documented bilateral macular degeneration and hypoplasia of the cerebellar vermis and corpus callosum, with persistence of cavum vergae, respectively. Lysosomal diseases, lipofuscinosis, organic acidurias, aminoacidopathies, biotidinase deficiency and endocrine disorders were excluded by laboratory examinations. Ultrastructural analyses of skin and muscle biopsies did not reveal significant alterations. High resolution karyotype was normal.

### Patient IV23

This patient, born in 1979 from non-consanguineous parents, showed moderate generalized hypotonia at birth, delayed psychomotor development, acquiring an unaided but unsteady gait at 4 years, and impaired speech. Since the first years of life, the patient suffered from recurrent bronchopulmonary infections. His neurological condition was similar to that of patient IV4, with generalized hypotonia, trunk and limb ataxia, dysmetria and hypoactive deep tendon reflexes. A few years later, drug-resistant seizures appeared and the ability to walk was lost, with a severe degeneration of cognitive functions. Brain CT-scan and MRI showed persistence of the cavum vergae, hypoplasia of cerebellar vermis and corpus callosum. Fundoscopy revealed bilateral macular degeneration. Cytogenetic studies with high resolution banding revealed a normal karyotype.

Affected males’ mothers (III1 and III10) appeared neurologically normal and their upper-limb X-rays showed Madelung deformity.

## Results

### Molecular analyses

As suggested by Des Portes et al. [8], who identified the disease locus (Xp22.33) in the present family, we analysed additional microsatellite markers mapping within the PAR1 to confirm linkage results, thus detecting a deletion in obligate carrier females (III1 and III10), III3, III7, IV2 and IV15 (data not shown).

XCI analysis revealed a skewed inactivation of X chromosome in the peripheral blood cells of all carrier females, with ratios of peak areas ranging from 97:3 to 85:15.

### FISH and array-CGH analyses

**Fig. 2:**
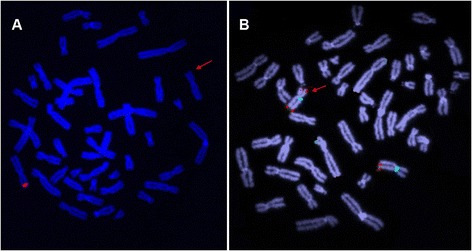
FISH analysis with Xp/Xq subtelomeric probes on peripheral blood metaphases of female IV2. Panel A shows the Xp deletion, with the *red arrow* indicating the region where the signal probe is absent. Panel B shows the recombinant X chromosome with two Xq probe signals. The X chromosome centromere is marked with a specific aqua alphoid DNA probe

**Fig. 3:**
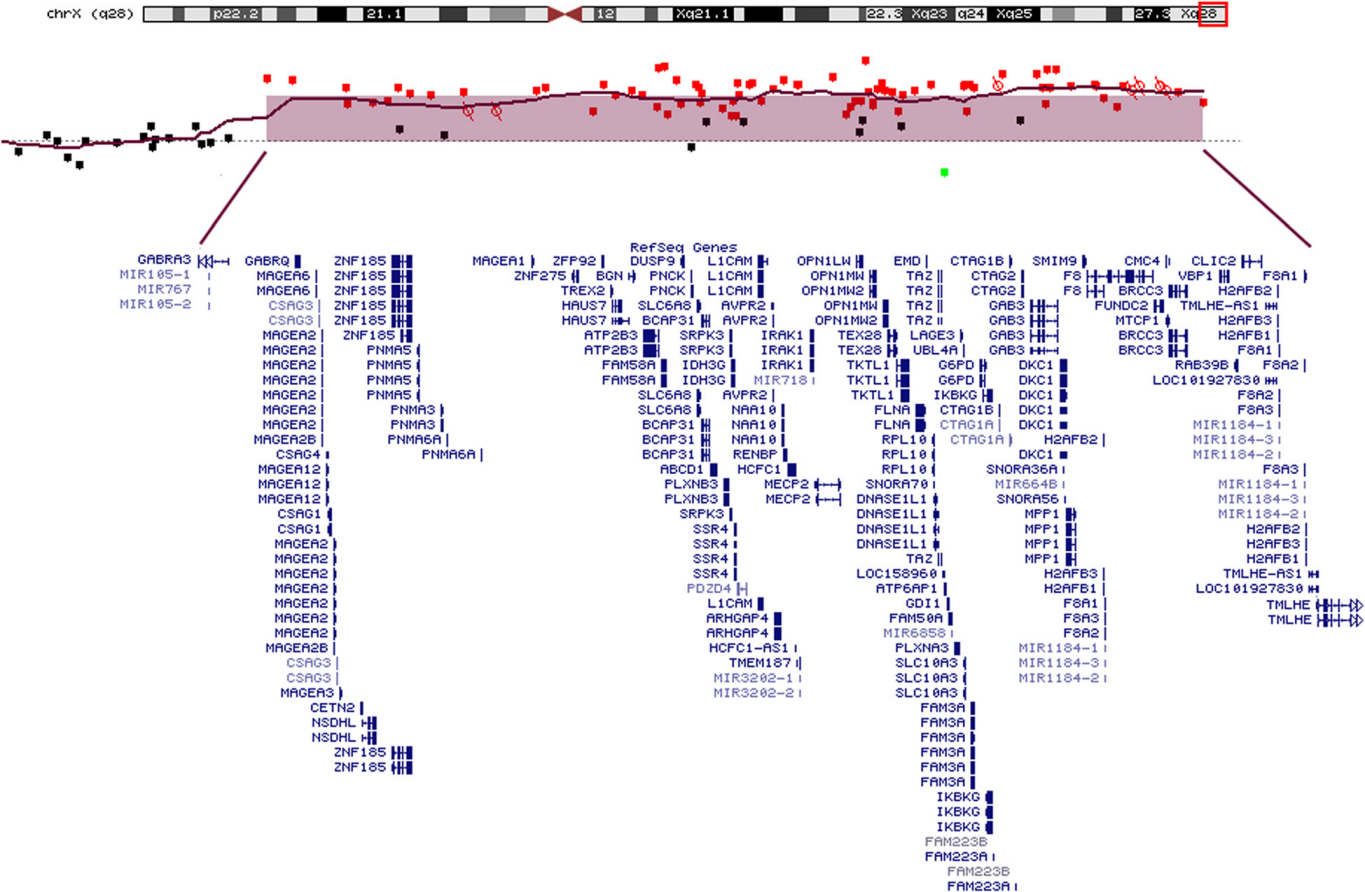
Xq28 duplication identified by array-CGH analysis. The duplicated region is circled on the X chromosome ideogram and the RefSeq genes involved are reported under the array-CGH profile

FISH analysis with XpYp subtelomere specific probes confirmed the microdeletion in the tip of chromosome X short arm (Fig. 2a) in all studied samples (III1, III3, III7, III10, IV2 and IV15). As the loss of PAR1 did not explain the neurologic phenotype observed in males, the DNA of carrier female IV2 was analysed by array-CGH to evaluate the presence of additional cryptic imbalances involving different genomic regions. Since both PAR regions were not covered by oligonucleotides in the microarray platform used, we were not able to see the deletion in Xp, but a terminal duplication in Xq28 was detected, encompassing nearly 3.3 Mb (chrX:151,532,990–154,841,455) and 130 genes, including *MECP2* (Fig. 3).

Additional FISH experiments were performed to define more precisely the breakpoints of the deletion in Xp and to determine the localization of the Xq duplicated segment. BAC, PAC and fosmid clones specific for PAR1 mapped the deletion breakpoint in a ~50 kb region in Xp22.33, between clones G248P87136C11 (1,097,568–1,134,076) and WI2 82904A1 (1,184,114–1,227,822) (Table 1). The deleted segment was therefore entirely located within the PAR1 region, extending about 1.2 Mb and containing five RefSeq genes (*PLCXD1*, *GTPBP6*, *LINC00685*, *PPP2R3B* and *SHOX*).

**Table 1:** List of FISH clones used to redefine the Xp deletion. Genomic position of probes is reported according to the February 2009 human reference sequence (GRCh37/hg19) and the presence or the absence of the corresponding signals on the normal and recombinant X chromosomes are indicated by + and − signs, respectively

Clone	Position bp (hg19)	X chromosome	X derivative
RP13 167H21	803878–917875	+	--
RP11 309 M23	915876–1047557	+	--
WI2 87136C11	1097568–1134076	+	--
WI2 829094A1	1184114–1227822	+	+
RP4 674 K6	1347892–1430773	+	+

Finally, a FISH experiment carried out by using an Xq subtelomeric probe showed that the Xq28 duplicated segment was translocated to the terminal p-arm region replacing the deleted portion (Fig. 2b).

## Discussion

In this study, cytogenetic and molecular cytogenetic analyses performed on the family previously described by Bertini et al. [7] revealed the presence of a recombinant X chromosome segregating with the disease trait.

Microsatellite markers analysis and FISH studies detected a 1.2 Mb terminal deletion of the short arm of chromosome X, including *SHOX*. The haploinsufficiency of this gene, which escapes lyonization, causes X-linked idiopathic short stature (MIM 300582) in Turner syndrome and Leri-Weill dyschondrosteosis (autosomal dominant; MIM 127300) whereas mutations of both alleles give rise to Langer mesomelic dysplasia (MIM 249700). These syndromes are characterized essentially by short stature and skeletal defects without neurological implications. Therefore, the *SHOX* gene deletion may be the cause of the Madelung deformity observed in carrier females, but the severe neurologic phenotype affecting males of this family is likely due to the Xq28 duplication identified through array-CGH analysis.

The 3.3 Mb duplicated segment encompasses *MECP2* gene, whose duplications are known to cause a defined syndrome with cardinal features that include early infantile hypotonia, delayed psychomotor development leading to severe to profound ID with absent to very limited speech and neurological symptoms (abnormal gait, epilepsy and spasticity) [9]. In addition, recurrent infections, especially of the respiratory tract, gastrointestinal motility problems and structural brain anomalies (in patients who underwent brain imaging) are frequently recorded.

Although duplications reported in the medical literature have variable sizes and include additional genes, neurological defects are mainly ascribed to dosage alteration of *MECP2*. Mice overexpressing this gene develop key neurological features of *MECP2* duplication syndrome [10, 11]. Moreover, the minimal duplicated region described to date in affected males involves exclusively *MECP2* and *IRAK1* and is sufficient to cause the core phenotype [12]. In addition, also in patients with chromosomal rearrangements leading to Xq28 functional disomy, clinical features are consistent with those of *MECP2* duplication syndrome, supporting further a major role of this gene in the manifestation of the disease [13]. Recently, the overexpression of *GDI1* and *RAB39B* has been proposed to cause cognitive impairment in patients carrying two different recurrent Xq28 duplications not involving *MECP2*, but this milder phenotype implies a modest contribution of dosage alterations of these genes to the severe clinical manifestations of *MECP2* duplication syndrome due to large aberrations [14, 15].

In the present family, all affected males showed generalized hypotonia since birth; those who deceased at younger age died of severe gastroenteritis (II4) and bronchopneumonia (III4, III5, III8 and IV3). The two elder patients (IV4 and IV23), in addition to congenital hypotonia and recurrent infections, presented other features ascribable to *MECP2* duplication syndrome, such as motor and mental deterioration with development of unsteady gait and impaired speech that were completely lost at 9 to 10 years, ataxia, seizures and hypoplasia of the cerebellar vermis and corpus callosum. Macular degeneration is an important additional clinical feature observed in the present family, but it has never been described in patients with *MECP2* duplications or recombinant X chromosomes involving more extended regions on both arms [6, 13].

Excluding a missed diagnosis of maculopathy in published cases, these findings would suggest that genes mapping within the rearranged portions of the X chromosome are not responsible for macular degeneration. However, maculopathy was reported in some families with X-linked blue cone monochromacy (MIM 303733) caused by deletions within the opsin genes locus in Xq28 [16–19], suggesting that dosage alterations of these genes could play a role in macular atrophy through an unknown mechanism that would need additional genetic or environmental factors to occur. In the present family, the expression of opsin genes (or other genes with a potential role in structural and functional integrity of the macula), included in the duplicated region, could be altered not only by the presence of extra copies but also by a positional effect due to their translocation on Xp. It is difficult to establish whether in other published cases carrying recombinant X chromosome the Xq28 duplicated segment was inserted exactly in the same locus we detected in Xp22.33, because they were identified through low resolution techniques and breakpoints were not defined further [20, 21].

Nevertheless, we cannot exclude the involvement of genes mapping outside the aberration, but still on the rearranged X chromosome. Indeed, the maculopathy has been ascertained in the two elder affected males, who are second degree maternal cousins and could have inherited the trait exclusively by their healthy mothers, suggesting an X-linked transmission as for the rest of the phenotype. However, the hypothesis of an additional gene alteration independent from the rearrangement is unlikely. Indeed, it should have segregated and caused uniquely macular degeneration in at least one of the eight healthy males of the family, unless the physiologic meiotic recombination between the two homologues in carrier females was hampered by the altered structure of the recombinant X chromosome.

Females with Xq28 duplications are usually asymptomatic because of an extreme or complete skewed inactivation of the rearranged chromosome. Nevertheless, a clinical phenotype similar to males has been described in females when the duplicated region remains functionally active due to its translocation to an autosome [9]. In the present family, carrier mothers had a skewed X chromosome inactivation and presented only mild skeletal anomalies of the upper limbs ascribable to the deletion of the PAR1 region, which escapes lyonization.

Recombinant X chromosomes can arise through two possible mechanisms. When the breakpoints are flanked by repeated sequences, an intramolecular recombination between them could occur during spermatogenesis [22]. Otherwise, the rearrangement could be generated by a recombination event between an X chromosome with a pericentric inversion and the normal homolog during female meiosis [6, 23]. In this family, we defined the breakpoints through FISH and array-CGH analyses and homologous repeated sequences were not present in these regions, making the pericentric inversion the most likely promoting mechanism. Unfortunately, we could not verify this hypothesis because all affected males inherited the delXp/dupXq aberration from their mothers and the first generation was not available.

## Conclusions

This study identifies the Xq28 duplication as the actual cause of the syndrome presented by the family described by Bertini et al. [7] and previously linked to Xp22.33-pter region by des Portes et al. [8]. Moreover, the overlap of neurologic features observed in the presented family with the core phenotype of Xq28 duplication syndrome is in line with previous suggestions of a major pathogenetic role of *MECP2* dosage alteration. Finally, additional cases are needed to evaluate a possible clinical association between *MECP2* duplication syndrome and macular degeneration, whose pathogenesis in the reported family is still unclear.

## Materials and methods

### Molecular analyses

Four microsatellite markers (DXYS28, DXY233, DXYS228, DXYS230) mapping within the pseudoautosomal region 1 (PAR1), not completely covered in the previously published linkage analysis [8], were analysed in 12 family members (Fig. 1).

DNA was extracted from blood samples using QIAamp DNA Mini Kit (Qiagen). Microsatellite markers were amplified by standard PCR protocols with 5′ fluorescent labeled primers. PCR products were run on an ABI prism 3730 sequencer (Applied Biosystems) and peak size was analysed using GeneMapper software (Applied Biosystems).

X-chromosome inactivation (XCI) study was performed on IV2 and on carrier females III1, III3 and III10, using a modified standard method [24].

### Fluorescence In Situ Hybridization (FISH) analysis

FISH analysis was performed on peripheral blood samples using subtelomere specific probes for both arms of the X chromosome (XpYp, clone 839D20, locus DXYS129 and XqYq, clone C8.2/1, locus DXYS61, Cytocell™ Technologies Ltd; TelVision™ XpYp, locus DXYS129 and XqYq, EST Cdy 16c07 GenBankZ43206, Vysis Abbott Laboratories), human genomic clones from RPCI BAC and PAC clones Libraries (RP13-167H21, RP11-309M3 and RP4-674K6) kindly supplied by Resources for Molecular Cytogenetics, University of Bari (Bari, Italy), and human female clones from the WIBR2 Fosmid Library (WI2 82904A1 and G248P87136C11) obtained from The Wellcome Trust Sanger Institute (Cambridge, UK). Subtelomeres probes were hybridized following manufacturers’ instructions. Bacterial cultures and DNA isolation for BAC/PAC and fosmid clones were performed according to the BAC/PAC miniprep protocol from the Resources for Molecular Cytogenetics University of Bari website and to Wellcome Trust Sanger Institute suggested protocols respectively. Probes were labelled with Biotin-16-dUTP (Roche) by nick translation, FISH procedures performed according to a standard protocol and probes detected with FITC-Streptavidin (Vector).

Slides were analysed on a Zeiss Axioplan 2 (Carl Zeiss, Göttingen, Germany) epifluorescence microscope and image analysis was performed on an IKAROS karyotyping and an ISIS FISH image analysis workstation (Metasystems, Altlussheim, Germany).

### Array Comparative Genomic Hybridization (Array CGH) analysis

The DNA of female IV2 was analysed by array-CGH through an Agilent 44K platform (Agilent Technologies), with a mean resolution of about 75 Kb, following manufacturer’s instructions. A graphical visualization of the results was provided by the Genomic Workbench software v.7.0 and aberrations were called by the ADM1 algorithm with a sensitivity threshold of 6.0. Genomic position of alterations was reported according to the February 2009 human reference sequence (GRCh37/hg19).

## Consent

Written informed consent was obtained from the patient for publication of this Case report. A copy of the written consent is available.

## Abbreviations

PAR1: Pseudoatosomal region
FISH: Fluorescence in situ hybridization
CGH: Comparative genomic hybridization
FoSTeS: Fork stalling and template switching
BAC: Bacterial artificial chromosome
PAC: P1-derived artificial chromosome
